# Development and Validation of a Nomogram for Mortality Prediction in Septic Patients with Prolonged or Chronic Critical Illness

**DOI:** 10.3390/diagnostics16121766

**Published:** 2026-06-08

**Authors:** Mikhail Ya. Yadgarov, Olga Yu. Rebrova, Levan B. Berikashvili, Petr A. Polyakov, Kristina K. Kadantseva, Alexey A. Yakovlev, Andrey V. Grechko, Valery V. Likhvantsev

**Affiliations:** 1 Federal Research and Clinical Center of Intensive Care Medicine and Rehabilitology, Moscow 107031, Russia; myadgarov@fnkcrr.ru (M.Y.Y.); lberikashvili@fnkcrr.ru (L.B.B.); p.polyakov@fnkcrr.ru (P.A.P.); kkadanceva@fnkcrr.ru (K.K.K.); ayakovlev@fnkcrr.ru (A.A.Y.); avgrechko@fnkcrr.ru (A.V.G.); 2 Pirogov Russian National Research Medical University, Moscow 117997, Russia; o.yu.rebrova@gmail.com; 3 Department of Anesthesiology and Intensive Care, I.M. Sechenov First Moscow State Medical University, Moscow 119991, Russia

**Keywords:** sepsis, chronic critical illness, intensive care unit, mortality prediction, nomogram, RICD

## Abstract

**Background/Objectives:** Patients with prolonged or chronic critical illness (PCI/CCI) represent a subgroup characterized by extended stays in an intensive care unit (ICU), persistent organ dysfunction, and increased susceptibility to recurrent sepsis episodes. Current sepsis prognostic tools have not been specifically tailored for this high-risk population. This study aimed to develop and validate a prognostic nomogram for predicting mortality in septic ICU patients with PCI/CCI. **Methods:** Data were obtained from the Russian Intensive Care Dataset (RICD). Eligible patients had confirmed sepsis episodes according to Sepsis-3 criteria. The cohort was randomly split into training and testing sets in a 7:3 ratio. Multivariable Cox regression identified predictors of mortality, which were incorporated into a prognostic nomogram. Predictive accuracy was assessed using Harrell’s C-index, and horizon-specific area under the receiver operating characteristic curve (AUROC). **Results:** A total of 336 septic patients were analyzed, with an overall ICU mortality of 14.0%. Median ICU length of stay was 44 days, and median time to sepsis onset was 10 days. Recurrent sepsis episodes occurred in 28.6% of patients. In multivariable analysis, four predictors of mortality were identified: age, SOFA score at sepsis onset, type 2 diabetes mellitus, and time to sepsis onset. The nomogram demonstrated a C-index of 0.787 (95% confidence interval [CI] 0.669; 0.890) in the training set and one of 0.715 (95% CI 0.584; 0.836) in the testing set. In the testing set, the horizon-specific AUROCs were 0.898, 0.741, and 0.703 for 14-, 28-, and 42-day survival prediction, respectively. **Conclusions:** The prognostic nomogram, specifically tailored for PCI/CCI septic patients, demonstrated a testing-set C-index of 0.715, with higher 14-day predictive performance, whereas predictive accuracy decreased at later time points. Prospective multicenter validation is necessary before clinical implementation.

## 1. Introduction

Sepsis, defined as life-threatening organ dysfunction resulting from a dysregulated host response to infection [1], remains a leading cause of morbidity and mortality in intensive care units (ICUs) worldwide [2]. Despite significant advances in early recognition, antimicrobial therapy, fluid resuscitation, and vasopressor support, sepsis continues to account for a substantial proportion of ICU admissions (ranging from 13.6% to 39.3% [3]) and contributes to high healthcare costs and long-term disability among survivors [4]. Mortality rates among septic ICU patients remain unacceptably high, reaching up to 50% in some cohorts, and are influenced by multiple factors, including age, baseline disease severity, comorbidities, and the prompt initiation of therapeutic interventions [5,6].

Timely and accurate risk stratification is essential to guide therapeutic decision-making and allocate intensive care resources effectively in this high-risk population [7]. Traditional scoring tools, such as the Nutrition Risk in the Critically Ill (NUTRIC), Acute Physiology and Chronic Health Evaluation II (APACHE II), and the Sequential Organ Failure Assessment (SOFA) score, are widely employed in clinical practice for severity assessment and mortality prediction [8,9]. However, these scoring systems often demonstrate suboptimal performance when applied to septic patients, and their clinical utility is limited by complex calculation methods and challenges in real-time dynamic monitoring [8].

In recent years, numerous mortality prediction models for patients with sepsis have been proposed, including those based on machine learning approaches [10]. However, all mortality prediction models were developed for general ICU or general ward populations, limiting their applicability to distinct patient subgroups. One such subgroup comprises patients with prolonged or chronic critical illness (PCI/CCI) [11]. Patients with PCI/CCI represent a specific subgroup, characterized by persistent organ dysfunction, prolonged ICU stays [11], and a markedly increased risk of adverse outcomes, including hospital-acquired infections and repeated episodes of sepsis [12]. Despite growing interest, a consensus definition for PCI/CCI has yet to be established [13], and no sepsis mortality prediction models have been specifically tailored or validated for this high-risk population.

To address this gap, the present study aimed to develop and validate a nomogram for predicting mortality in septic patients with PCI/CCI.

## 2. Materials and Methods

### 2.1. Source of Data

Data for this study were obtained from the Russian Intensive Care Dataset (RICD) v2.0 [14,15], a de-identified, high-resolution clinical repository maintained by the Federal Research and Clinical Center of Intensive Care Medicine and Rehabilitology (FRCC ICMR)—a major tertiary center specializing in the management and research of patients with PCI/CCI. The RICD contains comprehensive patient-level information, including anthropometric characteristics, continuously recorded vital signs, therapeutic interventions, laboratory test results, severity scores, and discharge summaries. The dataset comprises 3607 ICU admissions from 3404 distinct patients treated at FRCC ICMR between December 2017 and September 2024. The present study is a retrospective analysis based on the Russian Intensive Care Dataset (RICD). At the time of ethics approval, the study protocol referred to RICD version 1.0, which already included patients up to December 2023. Subsequently, the database was updated to version 2.0, which includes ICU admissions up to September 2024. Importantly, this update did not involve any changes to the study design, inclusion criteria, or variables analyzed, and therefore did not constitute a modification of the originally approved protocol. No prospective patient enrollment was performed; the study is based solely on secondary analysis of a de-identified dataset.

### 2.2. Study Design and Setting

A single-center retrospective cohort study using registry data was conducted. This real-world data study screened all ICU admissions recorded in the RICD. Eligible patients were those with an ICU length of stay ≥ 24 h and at least one sepsis assessment according to the Sepsis-3 criteria [16] during their ICU stay. Exclusion criteria were lack of continuously recorded vital signs, ICU readmissions, or absence of therapeutic intervention data. For patients with multiple ICU admissions, only the first admission was analyzed.

All eligible cases over the 8-year period were included; no sample size calculation was performed. The study was approved by the FRCC ICMR Local Ethics Committee (approval No. 1/24/1, 24 April 2024) and conducted in accordance with the Transparent Reporting of a multivariable prediction model for Individual Prognosis Or Diagnosis (TRIPOD) guidelines [17]. The TRIPOD checklist is provided in Supplementary Table S1.

### 2.3. Data Collection

Data extraction and preprocessing were conducted using DB Browser for SQLite v3.13.1 and Python v3.12, with all processing scripts publicly available on GitHub (https://github.com/MikhailYadgarov/RICDv2-sql-code, accessed on 9 January 2026). Extracted variables included the occurrence of sepsis and the timing of its onset; anthropometric characteristics (sex, age, body mass index); comorbidities; severity scores at ICU admission and at the onset of the sepsis episode 1; laboratory results and continuously recorded vital signs (heart rate, respiratory rate, body temperature, systolic, diastolic, and mean arterial blood pressure, oxygen saturation) at sepsis onset; and clinical outcomes, including all-cause mortality, septic shock, ICU and hospital length of stay, recurrent sepsis episodes and their duration, sepsis phenotypes, vasopressor and/or inotrope use, mechanical ventilation, and nosocomial pneumonia. The end of a sepsis episode was defined as the discontinuation of antibiotic therapy [18,19]. Given the heterogeneity of sepsis, episodes were classified as hyperinflammatory phenotype if a systemic inflammatory response syndrome (SIRS) score ≥ 2 was recorded at any time during the episode, and as hypoinflammatory otherwise. SIRS criteria were assessed retrospectively, whereas SOFA scores were directly retrieved from the electronic medical records. Given the retrospective nature of the study and the use of routinely collected electronic health records, no additional blinding procedures were required.

### 2.4. Statistical Analysis and Nomogram Development

Continuous data were presented as medians with interquartile ranges (IQR), and categorical data as absolute numbers and percentages. Comparisons between groups were performed using the Chi-square or Fisher’s exact test for categorical variables, and the Mann–Whitney U test (Wilcoxon rank-sum test) for continuous variables. All tests were two-sided, with a significance threshold of *p* < 0.05.

Survival time was measured from the onset of the sepsis episode 1 (left-truncation) to avoid immortal time bias. Univariate Cox proportional hazards regression was used to screen potential mortality prognostic factors, with variables showing significant associations and ≤30% missing data considered for multivariable analysis. No imputation of missing data was performed. Backward stepwise selection based on the likelihood ratio test was applied to derive the final multivariable model. The default stepwise criteria were used, with probability for entry of 0.05 and probability for removal of 0.10. The proportional hazards assumption for the final multivariable Cox model was assessed using Schoenfeld residuals.

A Cox regression-based nomogram was selected because the primary outcome was time-to-event mortality after sepsis onset, requiring consideration of survival time and censoring. For nomogram development, the study cohort was randomly split into training and testing sets in a 7:3 ratio. The model was fitted using the training set, and the same coefficients were applied to the testing set for validation. Mortality predictors and their estimated coefficients retained in the final Cox multivariable model were then transformed into a point-based scale and displayed as a graphical nomogram using the nomocox command in Stata 18.0 version 5.1.65, which converts Cox regression coefficients into points with adjustable ranges and tick marks according to developer recommendations [20]. The ranges of the individual ‘score’ scales and the overall ‘total score’ scale were generated automatically by the program through linear scaling of the estimated Cox coefficients into a point-based system, summation of these points, and transformation of the total score into outcome probability via the baseline hazard function [20]. All statistical analyses were performed in IBM SPSS Statistics 29 (IBM SPSS Statistics for Windows, Version 29.0.1, IBM Corp., Armonk, NY, USA), Stata 18 (StataCorp 2023, Stata Statistical Software: Release 18. StataCorp LLC, College Station, TX, USA), and Python v3.12.0. Python analyses utilized the ‘pandas’, ‘numpy’, ‘lifelines’, and ‘matplotlib’ packages.

### 2.5. Model Performance and Validation

The predictive performance of the nomogram was evaluated using Harrell’s concordance index (C-index) with 95% confidence intervals estimated by bootstrapping (1000 resamples). Discrimination was further assessed using horizon-specific ROC curve analysis for prespecified binary mortality endpoints at 14, 28, and 42 days after sepsis onset. AUROCs were compared using DeLong’s test with Bonferroni correction for multiple comparisons [21].

Calibration was assessed using calibration plots, calibration intercept, calibration slope, and Brier score. Confidence intervals were estimated using Wald estimates for calibration intercept and slope and bootstrap resampling for Brier score. Clinical utility was examined with decision curve analysis (DCA) across a range of threshold probabilities. Model performance in the testing set was additionally evaluated in patient subgroups stratified by sepsis phenotype (hyperinflammatory vs. hypoinflammatory).

## 3. Results

### 3.1. Patient Characteristics

**Figure 1:**
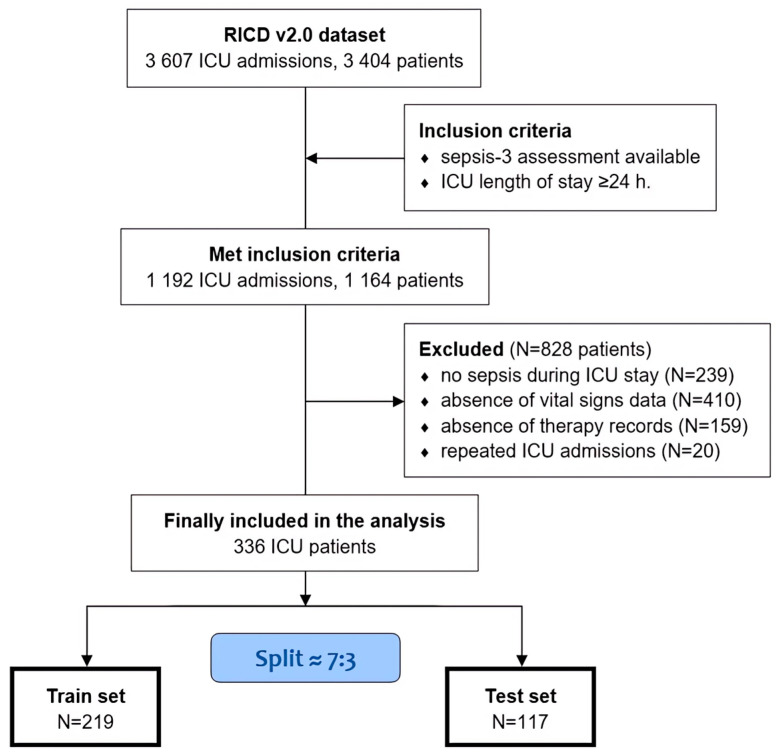
Flowchart of the study population selection.

After applying the eligibility criteria, 336 patients with sepsis were included in the analysis. Forty-seven patients (14.0%) died in the ICU, with a median time from the onset of the sepsis episode 1 to death of 36 days (IQR 27; 53). Kaplan–Meier survival curve is shown in Figure S1. The median age of the cohort was 64 years (IQR 48; 74). The study flow diagram is shown in Figure 1.

The median time from ICU admission to the onset of the sepsis episode 1 was 10 days (IQR 4; 17). The minimum ICU length of stay in the analyzed cohort was 24 days, and the median ICU length of stay of 44 days (IQR 30; 62). Therefore, all included patients fulfilled duration-based PCI/CCI definitions relying on ICU length of stay thresholds, including the commonly used cut-offs of ≥10 and ≥14 days [13]. Mechanical ventilation was required in 95% of patients, and more than 98% were transferred from external ICUs (Table 1). The most prevalent comorbidities were arterial hypertension (*n* = 269, 80.1%) and coronary artery disease (*n* = 209, 62.2%). Over 70% of patients presented with pneumonia at ICU admission.

A hyperinflammatory sepsis phenotype was observed in 243 patients (72.3%) during sepsis episode 1. Among non-survivors, septic shock during the first episode was observed more frequently among non-survivors (38.2% vs. 15.9%, *p* = 0.002), as was the requirement for vasopressors and/or inotropes (*p* < 0.001), and a sepsis episode 1 duration ≥ 21 days (*p* = 0.044). Recurrent sepsis episodes occurred in 96 patients (28.6%), The median time to recurrence from the end of the first episode was 9 days (IQR 4; 17). Mechanical ventilation was required during the sepsis episode 1 in 319 patients (94.9%).

Of the total cohort, 219 patients (65.2%) were randomly assigned to the training set and 117 patients (34.8%) to the testing set. The training and testing sets were comparable with respect to baseline characteristics and clinical outcomes (Table S2).

### 3.2. Screening for Predictive Factors

In univariate Cox regression analysis, significant predictors of ICU mortality included age (hazard ratio, HR 1.03, 95% CI 1.01; 1.05, *p* = 0.042), SOFA score at sepsis onset (HR 1.36, 95% CI 1.17; 1.58, *p* < 0.001), serum albumin (HR 0.83, 95% CI 0.70; 0.97, *p* = 0.022), total protein (HR 0.91, 95% CI 0.85; 0.98, *p* = 0.015), type 2 diabetes mellitus (HR 2.71, 95% CI 1.17; 6.26, *p* = 0.019), C-reactive protein (HR 1.006, 95% CI 1.001; 1.012, *p* = 0.016), time from ICU admission to the onset of the sepsis episode 1 (HR 1.001, 95% CI 1.001; 1.002, *p* = 0.036), and procalcitonin (HR 1.04, 95% CI 1.01; 1.08, *p* = 0.047).

In the multivariable Cox regression model four predictors were included after backward stepwise selection: SOFA score at sepsis onset (adjusted HR 1.30, 95% CI 1.12; 1.51, *p* < 0.001), time to sepsis onset (adjusted HR 1.001, 95% CI 1.001; 1.002, *p* = 0.011), type 2 diabetes mellitus (adjusted HR 2.26, 95% CI 0.94; 5.44, *p* = 0.068), and age (adjusted HR 1.03, 95% CI 0.99; 1.06, *p* = 0.081), while only SOFA and time to sepsis onset were statistically significant independent predictors (Table 2). Age and type 2 diabetes mellitus were retained in the final model by the backward likelihood-ratio selection procedure, although their *p*-values slightly exceeded the conventional 0.05 threshold. The proportional hazards assumption was not violated for any covariates or for the model globally (global test *p* = 0.92; all covariates *p* > 0.48; Table S3).

### 3.3. Mortality Prediction Nomogram Development and Validation

**Figure 2:**
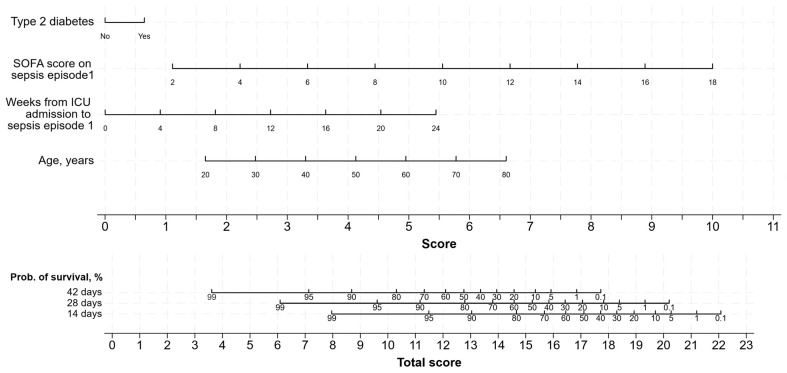
Nomogram to predict the probability of survival in septic ICU patients at three time points: 14, 28, and 42 days after sepsis onset. The “Score” axis represents unitless nomogram points assigned to each predictor. For each patient, points corresponding to age, time from ICU admission to sepsis episode 1, SOFA score at sepsis onset, and type 2 diabetes mellitus are summed to obtain the “Total score.” The total score is then projected onto the 14-, 28-, and 42-day survival probability scales to estimate the predicted probability of survival at each time point. Higher total scores correspond to lower predicted survival probabilities.

Based on multivariable Cox regression, a prognostic nomogram was developed, incorporating four predictors (Figure 2). This nomogram was subsequently validated using the testing set.

The C-index was 0.787 (95% CI 0.669; 0.890) for the training set, where the lower bound indicates low discriminative ability, and 0.715 (95% CI 0.584; 0.836) for the testing set. The model demonstrated acceptable performance in the hyperinflammatory phenotype (C-index 0.705, 95% CI 0.550–0.842), but showed inadequate predictive ability in the hypoinflammatory subgroup (C-index 0.674, 95% CI 0.367; 0.906).

**Figure 3:**
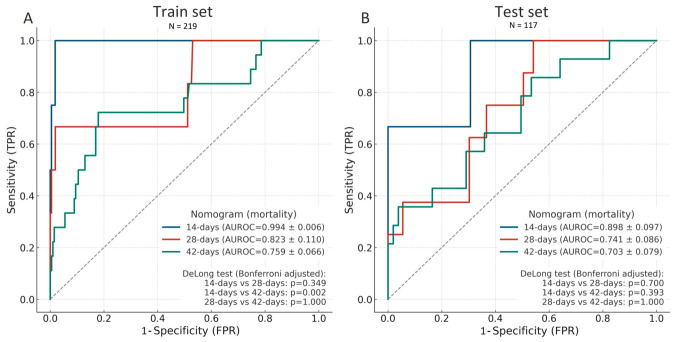
The horizon-specific ROC curves of the nomogram. (**A**) Train set; (**B**) Testing set.

The nomogram achieved AUROCs of 0.994 (95% CI 0.984; 1.000, 4 outcome events), 0.823 (95% CI 0.627; 1.000, 6 outcome events), and 0.759 (95% CI 0.629; 0.890, 18 outcome events) for predicting 14-, 28-, and 42-day survival in the training set, and 0.898 (3 outcome events), 0.741 (8 outcome events), and 0.703 (14 outcome events) in the testing set, demonstrating good predictive accuracy for 14-day prediction but limited performance for 28- and 42-day time points. Time-dependent ROC curves are shown in Figure 3.

**Figure 4:**
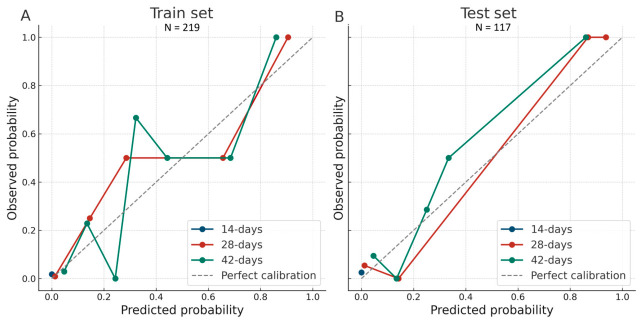
Calibration curves for the mortality risk prediction in septic patients. (**A**) Train set; (**B**) Testing set. For 14-day prediction, only a single point is displayed due to the small number of events (4 in the train set and 3 in the testing set).

Quantitative calibration assessment demonstrated generally acceptable weak calibration, although calibration varied across prediction horizons. In the testing set, calibration intercepts were 0.043 (95% CI −1.349; 1.435), 0.911 (95% CI 0.081; 1.741), and 0.169 (95% CI −0.466; 0.804) for 14-, 28-, and 42-day mortality prediction, respectively. Calibration slopes were 1.369 (95% CI 0.372; 2.367), 0.800 (95% CI 0.241; 1.360), and 0.657 (95% CI 0.100; 1.215), respectively. Brier scores were 0.011 (95% CI 0.000; 0.029), 0.050 (95% CI 0.017; 0.091), and 0.089 (95% CI 0.049; 0.135), respectively. These findings suggest acceptable calibration at 14 days, possible underestimation of 28-day mortality risk in the testing set, and less stable calibration at 42 days (Figure 4).

**Figure 5:**
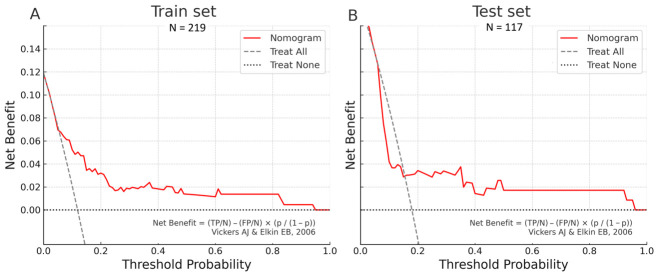
Decision curve analysis (DCA) for the mortality risk prediction in septic patients. (**A**) Train set; (**B**) Testing set [22].

Additionally, DCA showed positive net benefit in the testing set at threshold probabilities of 0.20 and 0.30, with net benefit values of 0.034 and 0.033, respectively (Figure 5).

## 4. Discussion

### 4.1. Key Findings

In this study, a prognostic nomogram was developed and validated to estimate the risk of mortality at 14, 28, and 42 days after the onset of a sepsis episode in ICU patients with PCI/CCI. The analysis included 336 patients (14.0% mortality) from the institutional RICD v2.0 database with a median ICU stay of 44 days and median time from ICU admission to sepsis onset of 10 days. The cohort was randomly split into training and testing sets in a 7:3 ratio, resulting in 219 and 117 patients, respectively. The final Cox proportional hazards multivariable model incorporated four predictors available at sepsis onset: SOFA score, age, type 2 diabetes mellitus, and time from ICU admission to sepsis onset. Model discrimination yielded a C-index of 0.787 in the training set and 0.715 in the testing set. Horizon-specific ROC analysis demonstrated high predictive accuracy for short-term outcomes (AUROC 0.994 at day 14 in the training set and 0.898 in the testing set), with reduced performance for 28- and 42-day predictions. The model showed better performance in patients with a hyperinflammatory phenotype (C-index 0.705) compared to those with a hypoinflammatory phenotype (C-index 0.674).

### 4.2. Relationship with Previous Studies

Several previous studies have used RICD v2.0 for prognostic modeling in patients with PCI/CCI. In our previous work, we developed a machine-learning model for predicting sepsis development in this population [23]. The present study represents a subsequent analysis of the septic PCI/CCI subgroup identified in the previous study [23], with a different endpoint, modeling objective, and statistical approach. In another related study, we evaluated the prognostic value of APACHE II and NUTRIC scores for mortality prediction in patients with chronic critical illness [24]. This previous cohort shares an exact overlap of 79 patients with the present septic population, although clinical variables, study objectives, and eligibility criteria remain entirely distinct. In the present study, we focused on short-term mortality prediction after sepsis onset in septic patients with PCI/CCI.

The findings of this study are generally consistent with previously published results. Higher SOFA score is a well-established predictor of mortality in sepsis [25,26]. Extended length of stay before developing sepsis was associated with higher mortality in our study. Kim, J.Y. et al. (2024) in a study of 1395 patients demonstrated that prolonged length of stay greater than five days before sepsis onset was associated with increased mortality risk (adjusted odds ratio 3.00 [95% CI 1.69; 5.34 [27]]). Older age is a well-known risk factor for sepsis mortality [28]. In a meta-analysis by Du, Q. et al. (2024) including 23 studies (14,521,791 septic patients), diabetes mellitus slightly increased overall sepsis mortality (relative risk 1.12 [95% CI 1.00; 1.25], *p* < 0.001) [29]. In the present model, age and type 2 diabetes mellitus were retained by the backward likelihood-ratio selection procedure, although their individual associations did not reach conventional statistical significance; therefore, their contribution should be interpreted in the context of the overall multivariable model.

In univariate analysis, APACHE II and NUTRIC scores were associated with mortality. In the study by Moubarez, D.A. (2023), involving 410 patients with sepsis (mortality 54.9%), APACHE II and NUTRIC were also statistically significant predictors of mortality (*p* < 0.001) [9]. Similarly, Hai, P.D. et al. (2022) analyzed 194 patients with sepsis (mortality 37.6%) and found that both NUTRIC (AUROC = 0.79) and APACHE II (AUROC = 0.78) were significant predictors of mortality [26]. However, both studies were conducted in general sepsis ICU populations with substantially higher mortality than in the present PCI/CCI cohort, which limits direct comparison of prognostic performance. Eosinopenia was also associated with increased mortality in the univariate analysis, consistent with previous reports [30,31]. Malnutrition is a recognized risk factor for mortality in sepsis [32]. In the present study, lower total protein and albumin levels in the univariate analysis were likewise associated with higher mortality.

The prognostic performance of the present nomogram is comparable with previously published nomogram-based models developed for specific septic ICU subgroups. Tong et al. developed a Cox regression nomogram for septic patients with heart failure using the MIMIC-IV database and predicted 7-, 15-, and 30-day survival based on 13 variables, including age, pneumonia, mechanical ventilation, laboratory parameters, SOFA score, and comorbidity burden [33]. Gu et al. developed a nomogram for 28-day mortality in septic patients with coronary artery disease using the MIMIC-III database; their model incorporated age, diastolic blood pressure, pH, lactate, red cell distribution width, anion gap, vascular comorbidities, and AKI stage, and showed better discrimination than SOFA alone [34]. Yang et al. constructed a nomogram for 28-day mortality prediction in sepsis patients using clinical data from two tertiary hospitals. Their logistic regression model incorporated five predictors: age, SOFA score, C-reactive protein, mechanical ventilation, and vasoactive drug use [35]. However, these studies focused on comorbidity-defined sepsis subgroups and used variables obtained at ICU admission, whereas our study incorporated variables measured at sepsis onset.

This approach may be particularly relevant for early risk assessment after sepsis onset. In the present study, AUROC values were numerically higher for 14-day prediction than for 28- and 42-day predictions; however, this observation should be interpreted cautiously because the 14-day estimates were based on a very small number of early mortality events. Moreover, the prolonged ICU length of stay in our cohort complicates direct comparisons with earlier models, which were predominantly developed in general ICU populations with shorter and less complex critical illness trajectories.

The prognostic performance of the present nomogram falls within the range reported for recently published sepsis mortality nomograms, although direct comparison is limited by differences in study populations, outcomes, sample sizes, and performance metrics. Gu et al. reported C-index values of 0.667 in the training set and 0.661 in the validation set, with corresponding AUC values of 0.719 and 0.724 [34]. Tong et al. reported C-index values of 0.730 in the training set and 0.761 in the testing set [33]. Yang et al. reported higher discrimination for 28-day mortality prediction in sepsis, with AUC values of 0.849 in the training set and 0.837 in the validation set [35]. The results should be interpreted in the context of the smaller sample size and the more specific PCI/CCI septic population analyzed in the present study.

Sepsis is a heterogeneous clinical syndrome with substantial biological and phenotypic variability. Depending on the classification method, previous studies have identified between two and four distinct sepsis phenotypes, each differing in immune response profiles, clinical trajectory, and outcomes [36,37,38]. In the present study, patients were stratified into two phenotypes according to SIRS criteria, with 243 (72%) sepsis episodes classified as hyperinflammatory. In the hypoinflammatory subgroup, the model did not achieve adequate predictive performance, which may be explained by the imbalance in phenotype distribution, the limited sample size, and the distinct clinical characteristics of these patients. Therefore, clinical application of the nomogram in patients with a hypoinflammatory sepsis phenotype cannot be recommended.

The relatively low ICU mortality should be interpreted in the context of the specific clinical trajectory of the cohort. Most patients were transferred from external ICUs and had therefore survived the earliest phase of critical illness before admission to our center. In contemporary sepsis cohorts characterized by subsequent development of CCI, early or in-hospital mortality has also been reported to be relatively low: 13% in a surgical ICU sepsis cohort (49% developed CCI) [39] and 6% in an abdominal sepsis cohort (37% developed CCI) [40]. Thus, the ICU mortality of 14.0% observed in our study is consistent with published data.

### 4.3. Significance of Study Findings

The developed nomogram provides a clinically applicable tool for estimating short-term mortality risk in patients with PCI/CCI at the onset of sepsis. By relying on four routinely measured parameters, the model enables rapid risk stratification in a PCI/CCI population that is typically underrepresented in sepsis prognostic research.

Based on DCA in the testing set, the most consistent positive net benefit was observed at threshold probabilities of 0.20–0.30, with the highest net benefit at 0.20. Therefore, a predicted mortality risk of approximately ≥20% may be considered a candidate threshold for identifying high-risk patients in future prospective studies. However, this threshold should not be interpreted as a definitive clinical decision threshold until externally validated and tested in prospective studies.

After prospective multicenter validation and impact analysis, such a model could potentially be integrated into ICU monitoring systems as an automated risk-stratification aid. However, its ability to improve clinical decision-making, escalation of care, or ICU resource allocation requires dedicated prospective evaluation and should be considered hypothesis-generating at this stage.

### 4.4. Strengths and Limitations

To our knowledge, this is the first study to develop and validate a mortality prediction model specifically tailored for ICU septic patients with PCI/CCI.

Nevertheless, several limitations should be considered when interpreting these findings.

First, the RICD v2.0 dataset is derived from a single-center ICUs, which may limit the generalizability of the findings. In addition, PCI/CCI is not a uniformly defined condition, and published definitions differ substantially, incorporating ICU length of stay, prolonged mechanical ventilation, tracheostomy, persistent organ dysfunction, or combinations of these criteria [13]. Because these definitions may identify clinically distinct patient populations, we did not classify patients according to a single PCI/CCI definition post hoc. This limits direct comparability with studies applying formal PCI/CCI definitions. Second, no formal sample size calculation was performed. Although the final model included 47 outcome events and 4 retained predictors, the resulting events-per-variable ratio was close to the lower boundary of commonly accepted requirements for prognostic modeling; therefore, the model estimates should be interpreted with caution. In addition, the two-stage variable selection strategy may have increased the risk of overfitting. Third, external validation was not performed. Fourth, the limited number of early mortality events restricted the interpretation of 14-day calibration, as reflected by the reduced number of informative calibration points at this prediction horizon. Fifth, the model was based solely on baseline variables measured at sepsis onset, without incorporating dynamic changes that may enhance long-term prognostic accuracy. Additionally, given the high proportion of missing data in several variables, restricting the multivariable analysis to statistically significant predictors with ≤30% missingness minimized the risk of model instability but may have limited the inclusion of clinically relevant covariates. Albumin was not assessed in a multiple-imputation sensitivity analysis because its missingness was substantial (53%), likely reflected selective laboratory testing in routine ICU practice, and would have introduced an additional post hoc model with limited stability in a cohort with few outcome events. Finally, SIRS-based sepsis phenotype stratification was used only as an exploratory approach to evaluate heterogeneity of model performance and was not intended to redefine sepsis phenotypes, which remain an area without universally accepted classification criteria. Our nomogram should not be applied to patients with a hypoinflammatory phenotype.

### 4.5. Future Prospects

This study highlights several key areas for future investigation. First, the establishment and open sharing of dedicated PCI/CCI datasets is essential to support external validation. Second, prospective multicenter studies are warranted to assess the generalizability and clinical utility of the proposed nomogram in real-world ICU settings. Third, the absence of a standardized definition for PCI/CCI remains a major barrier to cross-study comparisons.

## 5. Conclusions

The prognostic nomogram, specifically tailored for PCI/CCI septic patients, demonstrated acceptable overall discrimination in the testing set (C-index 0.715) and 14-day predictive performance, although the latter estimate was based on very few early outcome events, whereas predictive accuracy decreased at later time points. The model’s results emphasize the need for population-specific prognostic tools and highlight the potential of simple, routinely available parameters for rapid risk stratification in complex ICU cohorts. Further prospective multicenter studies are warranted to validate these findings.

## Figures and Tables

**Table 1:** Comparison of baseline characteristics and outcomes of septic intensive care unit patients.

Parameters		Survival Group *N* = 289	Death Group *N* = 47	*p*-Value
Time to sepsis onset, h		233 (111; 382)	249 (89; 573)	0.3 ^1^
Sepsis phenotype	Hyperinflammatory	204, 70.6%	39, 83.0%	0.08 ^2^
	Hypoinflammatory	85, 29.4%	8, 17.0%	
Sex	M	163, 56.4%	27, 57.4%	0.9 ^3^
	F	126, 43.6%	20, 42.6%	
Age, years		62 (48; 73)	72 (61; 79)	0.001 ^1^
BMI, kg/m^2^		*N* = 244, 24.8 (22.1; 29.4)	*N* = 40, 25.2 (21.8; 28.3)	0.6 ^1^
Transfer from another hospital		285, 98.6%	45, 95.7%	0.2 ^2^
Pneumonia on admission		206, 71.3%	37, 78.4%	0.3 ^3^
*Scale scores on admission*				
APACHE II, score		*N* = 73, 15 (12; 18)	*N* = 11, 21 (15; 25)	0.022 ^1^
NUTRIC, score		*N* = 73, 4 (3; 5)	*N* = 11, 5 (4; 7)	0.019 ^1^
SOFA, score		*N* = 285, 4 (3; 5)	*N* = 45, 4 (3; 6)	0.2 ^1^
SIRS, score		1 (1; 2)	1 (1; 2)	0.3 ^1^
FOUR, score		*N* = 268, 13 (10.5; 15)	*N* = 43, 13 (10; 14)	0.3 ^1^
GCS, score		*N* = 277, 11 (9; 13)	*N* = 45, 10 (8; 12)	0.2 ^1^
CRS-R, score		*N* = 178, 12 (6; 17)	*N* = 29, 11 (6; 17)	0.9 ^1^
*Scale scores on sepsis episode 1*				
APACHE II, score		*N* = 20, 16 (13.5; 17)	*N* = 4, 18 (14; 22)	0.6 ^1^
NUTRIC, score		*N* = 20, 4 (3; 5)	*N* = 4, 4 (4; 5.5)	0.6 ^1^
SOFA, score		5 (4; 6)	6 (4; 8)	0.001 ^1^
SIRS, score		1 (1; 2)	1 (1; 2)	0.1 ^1^
FOUR, score		*N* = 35, 13 (10; 16)	*N* = 7, 13 (9; 15)	0.7 ^1^
GCS, score		*N* = 47, 11 (9; 12)	*N* = 7, 11 (6; 11)	0.4 ^1^
CRS-R, score		*N* = 22, 17 (11; 20)	*N* = 4, 16.5 (10; 18)	0.5 ^1^
*Laboratory parameters on sepsis episode 1*				
Hemoglobin, g/L		*N* = 208, 99 (90; 109)	*N* = 37, 92 (83; 102)	0.055 ^1^
Leukocytes, 10^9^/L		*N* = 200, 9.3 (7.1; 12.3)	*N* = 33, 10.3 (7.1; 13.5)	0.4 ^1^
Neutrophils, 10^9^/L		*N* = 200, 1.4 (1.0; 1.9)	*N* = 34, 1.4 (0.9; 1.9)	0.9 ^1^
Lymphocytes, 10^9^/L		*N* = 200, 7 (5; 9)	*N* = 33, 8 (5; 11)	0.2 ^1^
Neutrophil-to-lymphocyte ratio		*N* = 200, 4 (3; 8)	*N* = 33, 6 (3; 11)	0.4 ^1^
Platelets, 10^9^/L		*N* = 200, 280 (208; 370)	*N* = 34, 241 (147; 322)	0.02 ^1^
Lactate, mmol/L		*N* = 46, 1.2 (0.8; 1.5)	*N* = 17, 1.2 (1.0; 1.8)	0.3 ^1^
Creatinine, μmol/L		*N* = 193, 75 (60; 106)	*N* = 35, 96 (53; 124)	0.5 ^1^
C-reactive protein, mg/L		*N* = 185, 58 (29; 120)	*N* = 33, 74 (38; 168)	0.2 ^1^
Albumin, g/L		*N* = 133, 27.5 (23.9; 30.6)	*N* = 25, 23.4 (22.0; 26.6)	0.002 ^1^
Eosinophiles, 10^9^/L		*N* = 200, 0.2 (0.1; 0.3)	*N* = 34, 0.1 (0.0; 0.1)	<0.001 ^1^
Total protein, g/L		*N* = 188, 56.4 (52.1; 61.3)	*N* = 34, 51.3 (46.7; 56.3)	<0.001 ^1^
Procalcitonin, ng/mL		*N* = 21, 0.3 (0.2; 0.9)	*N* = 10, 2.7 (0.3; 29.6)	0.053 ^1^
D-dimer, mg/L		*N* = 8, 2.6 (1.4; 7.2)	*N* = 2, 3.5 (1.1; 5.9)	0.7 ^1^
pH of arterial blood		*N* = 48, 7 (7; 8)	*N* = 17, 8 (7; 8)	0.9 ^1^
*Vital parameters on sepsis episode 1*				
Heart rate, per min		*N* = 281, 84 (74; 92)	*N* = 47, 82 (71; 91)	0.9 ^1^
Respiratory rate, per min		*N* = 190, 17 (17; 18)	*N* = 33, 17 (17; 18)	0.7 ^1^
Body temperature, C		*N* = 280, 37 (37; 37)	*N* = 45, 37 (37; 37)	0.4 ^1^
Systolic blood pressure, mm Hg		*N* = 286, 122 (108; 135)	*N* = 47, 129 (108; 139)	0.4 ^1^
Diastolic blood pressure, mm Hg		*N* = 286, 73 (65; 83)	*N* = 47, 73 (63; 81)	0.5 ^1^
Mean blood pressure, mm Hg		*N* = 208, 94 (84; 107)	*N* = 37, 98 (88; 105)	0.5 ^1^
SpO_2_, %		*N* = 277, 99 (98; 99)	*N* = 46, 99 (96; 99)	0.2 ^1^
*Comorbidity*				
Ischaemic stroke		131, 45.3%	22, 46.8%	0.9 ^3^
Haemorrhagic stroke		59, 20.4%	7, 14.9%	0.4 ^2^
Traumatic brain injury		55, 19.0%	9, 19.1%	0.9 ^2^
Type 2 diabetes mellitus		40, 13.8%	13, 27.7%	0.02 ^3^
Chronic kidney disease		36, 12.5%	10, 21.3%	0.1 ^3^
Chronic obstructive pulmonary disease		11, 3.8%	1, 2.1%	0.9 ^2^
Coronary artery disease		175, 60.6%	34, 72.3%	0.1 ^3^
Arterial hypertension		229, 79.2%	40, 85.1%	0.4 ^3^
Heart failure		55, 19.0%	10, 21.3%	0.7 ^3^
*Outcomes and complications*				
^# †^ Septic shock		42/264, 15.9%	13/34, 38.2%	0.002 ^3^
Recurrent septic episodes		82, 28.4%	14, 29.8%	0.8 ^3^
Number of septic episodes		1 (1; 2), from 1 to 4	1 (1; 2), from 1 to 5	0.8
Duration of sepsis episode 1, days		21 (11; 35)	28 (15; 34)	0.3
Duration of sepsis episode 1 ≥ 21 days		145, 50.2%	31, 66.0%	0.044 ^3^
Duration of all septic episodes, days		27 (16; 41)	32 (15; 38)	0.6
ICU length of stay, days		44 (29; 61)	42 (35; 66)	0.4
ICU length of stay after episode 1, days		33 (22; 50)	33 (23; 53)	0.8
Total hospital length of stay, days		59 (42; 71)	*N* = 47, 48 (35; 77)	0.3
^#^ Nosocomial pneumonia		255, 88.2%	44, 93.6%	0.4 ^2^
^#^ Need for mechanical ventilation		275, 95.2%	43, 91.5%	0.3 ^2^
^#^ Use of vasopressors/inotropes		67, 23.2%	26, 55.3%	<0.001 ^3^

Abbreviations: APACHE II, Acute Physiology and Chronic Health Evaluation II; NUTRIC, Nutrition Risk in the Critically Ill; CRS-R, Coma Recovery Scale-Revised; FOUR, Full Outline of UnResponsiveness; IQR, Interquartile Range; SIRS, Systemic Inflammatory Response Syndrome; SOFA, Sequential Organ Failure Assessment; MV, Mechanical Ventilation; BMI, Body Mass Index; ICU, Intensive Care Unit; GCS, Glasgow Coma Scale. ^1^ Mann–Whitney U-test; ^2^ Chi-square test; ^3^ Fisher’s Exact test. Continuous variables are presented as median (Q1; Q3); the number of patients (*N*) is provided when missing data were present. ^#^ During sepsis episode 1. ^†^ Patients receiving vasopressors prior to the onset of sepsis episode 1 were excluded (fulminant sepsis).

**Table 2:** Univariate and multivariable Cox regression analyses (train set).

Parameter	*N*	Missing, %	Univariate Analysis			Multivariable Analysis		
			HR	95% CI	*p*-Value	adj. HR	95% CI	*p*-Value
^#^ Age	219	0	1.03	1.01; 1.05	**0.042**	1.03	0.99; 1.06	0.081
^#^ SOFA (sepsis episode 1)	219	0	1.36	1.17; 1.58	**<0.001**	1.30	1.12; 1.51	**<0.001**
Albumin	102	53	0.83	0.70; 0.97	**0.022**	-	-	-
Total protein	140	36	0.91	0.85; 0.98	**0.015**	-	-	-
^#^ Type 2 diabetes mellitus	219	0	2.71	1.17; 6.26	**0.019**	2.26	0.94; 5.44	0.068
C-reactive protein	140	36	1.006	1.001; 1.012	**0.016**	-	-	-
^#^ Time to sepsis onset	219	0	1.001	1.001; 1.002	**0.036**	1.001	1.001; 1.002	**0.011**
Procalcitonin	17	92	1.04	1.01; 1.08	**0.047**	-	-	-

Abbreviations: HR, hazard ratio; CI, confidence interval; SOFA, Sequential Organ Failure Assessment. ^#^ Variables included in the multivariable analysis and retained in the final model after backward stepwise selection based on the likelihood ratio test.

## Data Availability

The RICD can be obtained upon request at https://fnkcrr-database.ru/. Source code available at GitHub (https://github.com/MikhailYadgarov/RICDv2-sql-code) and upon reasonable request.

## Supplementary Materials

The following supporting information can be downloaded at: https://www.mdpi.com/article/10.3390/diagnostics16121766/s1, Table S1. TRIPOD Checklist: Prediction Model Development and Validation; Table S2. Comparison of train and test sets; Table S3. Test of proportional hazards assumption (Schoenfeld residuals); Figure S1. Kaplan–Meier survival curve (all patients).

## Abbreviations

The following abbreviations are used in this manuscript:

APACHE II	Acute Physiology and Chronic Health Evaluation II
AUROC	area under the receiver operating characteristic curve
BMI	body mass index
CCI	chronic critical illness
CRP	C-reactive protein
CRS-R	Coma Recovery Scale-Revised
DCA	decision curve analysis
FOUR	Full Outline of UnResponsiveness
GCS	Glasgow Coma Scale
HR	hazard ratio
ICU	intensive care unit
IQR	interquartile range
NUTRIC	nutrition risk in critically ill
PCI	prolonged critical illness
RICD	Russian intensive care dataset
SAPS II	simplified acute physiology score II
SIRS	systemic inflammatory response syndrome
SOFA	sequential organ failure assessment
