# Supplementary material for: Development and Validation of a Nomogram for Mortality Prediction in Septic Patients with Prolonged or Chronic Critical Illness
Source: Diagnostics (Basel). 2026 Jun 8;16(12):1766. doi: 10.3390/diagnostics16121766 (PMC13298199; doi:10.3390/diagnostics16121766)
Supplement: Supplementary file 1 [file diagnostics-16-01766-s001.zip › diagnostics-4304444-supplementary.pdf]

Supplementary Table S1. TRIPOD Checklist: Prediction Model Development and Validation.

Section/Topic	Item		Checklist Item	Page
**Title and abstract**				
Title	1	D;V	Identify the study as developing and/or validating a multivariable prediction model, the target population, and the outcome to be predicted.	1
Abstract	2	D;V	Provide a summary of objectives, study design, setting, participants, sample size, predictors, outcome, statistical analysis, results, and conclusions.	1
**Introduction**				
Background and objectives	3a	D;V	Explain the medical context (including whether diagnostic or prognostic) and rationale for developing or validating the multivariable prediction model, including references to existing models.	2-3
	3b	D;V	Specify the objectives, including whether the study describes the development or validation of the model or both.	3
**Methods**				
Source of data	4a	D;V	Describe the study design or source of data (e.g., randomized trial, cohort, or registry data), separately for the development and validation data sets, if applicable.	3-4
	4b	D;V	Specify the key study dates, including start of accrual; end of accrual; and, if applicable, end of follow-up.	3-4
Participants	5a	D;V	Specify key elements of the study setting (e.g., primary care, secondary care, general population) including number and location of centres.	3-4
	5b	D;V	Describe eligibility criteria for participants.	3-4
	5c	D;V	Give details of treatments received, if relevant.	NA
Outcome	6a	D;V	Clearly define the outcome that is predicted by the prediction model, including how and when assessed.	4-5
	6b	D;V	Report any actions to blind assessment of the outcome to be predicted.	4
Predictors	7a	D;V	Clearly define all predictors used in developing or validating the multivariable prediction model, including how and when they were measured.	4
	7b	D;V	Report any actions to blind assessment of predictors for the outcome and other predictors.	4
Sample size	8	D;V	Explain how the study size was arrived at.	3-4
Missing data	9	D;V	Describe how missing data were handled (e.g., complete-case analysis, single imputation, multiple imputation) with details of any imputation method.	4-5
Statistical analysis methods	10a	D	Describe how predictors were handled in the analyses.	4-5
	10b	D	Specify type of model, all model-building procedures (including any predictor selection), and method for internal validation.	4-5
	10c	V	For validation, describe how the predictions were calculated.	5
	10d	D;V	Specify all measures used to assess model performance and, if relevant, to compare multiple models.	5-6
	10e	V	Describe any model updating (e.g., recalibration) arising from the validation, if done.	NA
Risk groups	11	D;V	Provide details on how risk groups were created, if done.	NA
Development vs. validation	12	V	For validation, identify any differences from the development data in setting, eligibility criteria, outcome, and predictors.	5-6
**Results**				
Participants	13a	D;V	Describe the flow of participants through the study, including the number of participants with and without the outcome and, if applicable, a summary of the follow-up time. A diagram may be helpful.	Fig 1
	13b	D;V	Describe the characteristics of the participants (basic demographics, clinical features, available predictors), including the number of participants with missing data for predictors and outcome.	Tab 1
	13c	V	For validation, show a comparison with the development data of the distribution of important variables (demographics, predictors and outcome).	Suppl ement
Model development	14a	D	Specify the number of participants and outcome events in each analysis.	6-7
	14b	D	If done, report the unadjusted association between each candidate predictor and outcome.	Tab 2
Model specification	15a	D	Present the full prediction model to allow predictions for individuals (i.e., all regression coefficients, and model intercept or baseline survival at a given time point).	Fig 2
	15b	D	Explain how to the use the prediction model.	7-8
Model performance	16	D;V	Report performance measures (with CIs) for the prediction model.	Tab 2, Fig 3-5
Model-updating	17	V	If done, report the results from any model updating (i.e., model specification, model performance).	NA
**Discussion**				
Limitations	18	D;V	Discuss any limitations of the study (such as nonrepresentative sample, few events per predictor, missing data).	10-11
Interpretation	19a	V	For validation, discuss the results with reference to performance in the development data, and any other validation data.	8-10
	19b	D;V	Give an overall interpretation of the results, considering objectives, limitations, results from similar studies, and other relevant evidence.	8-10
Implications	20	D;V	Discuss the potential clinical use of the model and implications for future research.	10-11
**Other information**				
Supplementary information	21	D;V	Provide information about the availability of supplementary resources, such as study protocol, Web calculator, and data sets.	11
Funding	22	D;V	Give the source of funding and the role of the funders for the present study.	11

*Items relevant only to the development of a prediction model are denoted by D, items relating solely to a validation of a prediction model are denoted by V, and items relating to both are denoted D;V. We recommend using the TRIPOD Checklist in conjunction with the TRIPOD Explanation and Elaboration document.

Table S2. Comparison of train and test sets.

Parameters		Train set N = 219	Test set N = 117	p-value
Time to sepsis episode 1 onset, h.		254 (117; 408)	193 (88; 382)	0.1[1]
Sepsis phenotype	Hyperinflammatory	155, 70.8%	88, 75.2%	0.4[3]
	Hypoinflammatory	64, 29.2%	29, 24.8%	
Sex	M	119, 54.3%	71, 60.4%	0.3[3]
	F	100, 45.7%	46, 39.3%	
Age, years		65 (48; 75)	62 (49; 73)	0.3[1]
BMI, kg/m2		N = 185, 24.9 (22.2; 29.3)	N = 99, 24.2 (21.9; 28.4)	0.5[1]
Transfer from another hospital		214, 97.7%	116, 99.1%	0.7[2]
Pneumonia on admission		153, 69.9%	90, 76.9%	0.2[3]
Scale scores on admission				
APACHE II, score		N = 57, 16 (13; 19)	N = 27, 15 (11; 18)	0.4[1]
NUTRIC, score		N = 57, 4 (3; 5)	N = 27, 4 (3; 5)	0.5[1]
SOFA, score		N = 215, 4 (3; 5)	N = 115, 4 (3; 5)	0.9[1]
SIRS, score		1 (1; 2)	1 (1; 2)	0.2[1]
FOUR, score		N = 203, 13 (10; 15)	N = 108, 12.5 (10; 15)	0.6[1]
GCS, score		N = 210, 11 (9; 13)	N = 112, 11 (9; 13)	0.8[1]
CRS-R, score		N = 132, 12 (6; 17)	N = 75, 12 (6; 18)	0.7[1]
Scale scores on sepsis episode 1				
APACHE II, score		N = 15, 17 (15; 23)	N = 9, 15 (13; 16)	0.06[1]
NUTRIC, score		N = 15, 4 (3; 5)	N = 9, 4 (4; 4)	0.5[1]
SOFA, score		5 (4; 6)	5 (4; 6)	0.6[1]
SIRS, score		1 (1; 2)	1 (1; 2)	0.9[1]
FOUR, score		N = 26, 13 (10; 16)	N = 16, 13 (11.5; 15)	0.4[1]
GCS, score		N = 37, 11 (9; 14)	N = 17, 11 (9; 11)	0.4[1]
CRS-R, score		N = 15, 17 (11; 21)	N = 11, 17 (11; 19)	0.8[1]
Laboratory parameters on sepsis episode 1				
Hemoglobin, g/l		N = 158, 98 (89; 109)	N = 87, 97 (88; 105)	0.6[1]
Leukocytes, 10^9/L		N = 151, 9.3 (7.0; 12.3)	N = 82, 9.3 (7.7; 13.3)	0.8[1]
Neutrophils, 10^9/L		N = 151, 1.4 (1.0; 1.9)	N = 83, 1.5 (0.9; 2.0)	0.8[1]
Lymphocytes, 10^9/L		N = 151, 7 (5; 10)	N = 82, 7 (5; 10)	0.9[1]
Neutrophil-to-lymphocyte ratio		N = 151, 4 (3; 8)	N = 82, 5 (3; 8)	0.8[1]
Platelets, 10^9/L		N = 151, 277 (209; 370)	N = 83, 272 (170; 354)	0.3[1]
Lactate, mmol/l		N = 33, 1.1 (0.8; 1.4)	N = 30, 1.3 (0.9; 1.8)	0.4[1]
Creatinine, µmol/L		N = 146, 75 (55; 106)	N = 82, 77 (63; 110)	0.6[1]
C-reactive protein, mg/l		N = 140, 63 (33; 130)	N = 78, 57 (28; 125)	0.5[1]
Albumin, g/l		N = 102, 26.9 (23.4; 30.3)	N = 56, 26.3 (22.4; 30.5)	0.4[1]
Eosinophiles, 10^9/L		N = 151, 0.1 (0.0; 0.3)	N = 83, 0.1 (0.1; 0.3)	0.3[1]
Total protein, g/l		N = 140, 55.0 (50.8; 60.5)	N = 82, 56.7 (51.4; 60.2)	0.6[1]
Procalcitonin, ng/ml		N = 17, 0.9 (0.3; 2.2)	N = 14, 0.3 (0.1; 4.7)	0.2[1]
D-dimer, mg/l		N = 7, 3.4 (1.4; 6.5)	N = 3, 1.4 (1.1; 7.9)	0.7[1]
pH of arterial blood		N = 35, 7 (7; 8)	N = 30, 7 (7; 8)	0.7[1]
Vital parameters on sepsis episode 1				
Heart rate, per min		N = 212, 84 (74; 92)	N = 116, 80 (72; 92)	0.2[1]
Respiratory rate, per min		N = 147, 17 (17; 18)	N = 76, 17 (17; 18)	0.1[1]
Body temperature, C		N = 210, 37 (37; 37)	N = 115, 37 (37; 37)	0.6[1]
Systolic blood pressure, mm Hg		N = 217, 121 (108; 134)	N = 116, 125 (111; 140)	0.1[1]
Diastolic blood pressure, mm Hg		N = 217, 73 (63; 82)	N = 116, 73 (66; 84)	0.5[1]
Mean blood pressure, mm Hg		N = 165, 93 (84; 102)	N = 80, 98 (85; 109)	0.08[1]
SpO2, %		N = 209, 99 (98; 99)	N = 114, 99 (98; 99)	0.3[1]
Comorbidity				
Ischaemic stroke		100, 45.7%	53, 45.3%	0.9[3]
Haemorrhagic stroke		36, 16.4%	30, 25.6%	0.043[3]
Traumatic brain injury		42, 19.2%	22, 18.8%	0.9[3]
Type 2 diabetes mellitus		36, 16.4%	17, 14.5%	0.8[3]
Chronic kidney disease		25, 11.4%	21, 17.9%	0.1[3]
Chronic obstructive pulmonary disease		11, 5.0%	1, 0.9%	0.064[2]
Coronary artery disease		141, 64.4%	68, 58.1%	0.3[3]
Arterial hypertension		175, 79.9%	94, 80.3%	0.9[3]
Heart failure		48, 21.9%	17, 14.5%	0.1[3]
Outcomes and complications				
All-cause mortality		26, 11.9%	21, 17.9%	0.1[3]
[#†]Septic shock		30/193, 15.5%	25/105, 23.8%	0.08[3]
Recurrent septic episodes		59, 26.9%	37, 31.6%	0.4[3]
Number of septic episodes		1 (1; 2)	1 (1; 2)	0.4[1]
Duration of sepsis episode 1, days		20 (11; 36)	23 (13; 33)	0.5[1]
Duration of all septic episodes, days		26 (15; 41)	30 (18; 40)	0.4[1]
Hyper- to Hypoinflammatory phenotype transition		10, 4.6%	8, 6.8%	0.5[2]
Hypo- to Hyperinflammatory phenotype transition		16, 7.3%	8, 6.8%	0.9[2]
ICU length of stay, days		43 (30; 62)	44 (33; 62)	0.7[1]
ICU length of stay after episode 1, days		33 (21; 50)	34 (23; 50)	0.4[1]

Total hospital length of stay, days	58 (38; 71)	57 (44; 73)	0.6[1]
#Nosocomial pneumonia	191, 87.2%	108, 92.3%	0.2[2]
#Need for mechanical ventilation	204, 93.2%	114, 97.4%	0.1[2]
#Use of vasopressors/inotropes	56, 25.6%	37, 31.6%	0.3[3]

Abbreviations: APACHE II, Acute Physiology and Chronic Health Evaluation II; NUTRIC, Nutrition Risk in the Critically Ill; CRS-R, Coma Recovery Scale-Revised; FOUR, Full Outline of UnResponsiveness; IQR, Interquartile Range; SIRS, Systemic Inflammatory Response Syndrome; SOFA, Sequential Organ Failure Assessment; MV, Mechanical Ventilation; BMI, Body Mass Index; ICU, Intensive Care Unit; GCS, Glasgow Coma Scale.

[1]Mann-Whitney U-test; [2]Chi-square test; [3]Fisher's Exact test.

Continuous variables are presented as median (Q1; Q3); the number of patients (N) is provided when missing data were present.

#During sepsis episode 1.

†Patients receiving vasopressors prior to the onset of sepsis episode 1 were excluded (fulminant sepsis).

Table S3. Test of proportional hazards assumption (Schoenfeld residuals).

Covariate	ρ	χ^2	df	p-value
Age	-0.012	0.01	1	0.938
Type 2 diabetes mellitus	-0.081	0.17	1	0.680
SOFA (sepsis episode 1)	-0.129	0.50	1	0.481
Time to sepsis episode 1 onset	0.057	0.07	1	0.798
Global test		0.96	4	0.915

Note: Schoenfeld residuals test for proportional hazards assumption. No violations were detected for individual covariates or for the model globally.

Figure S1. Kaplan–Meier survival curve (all patients).

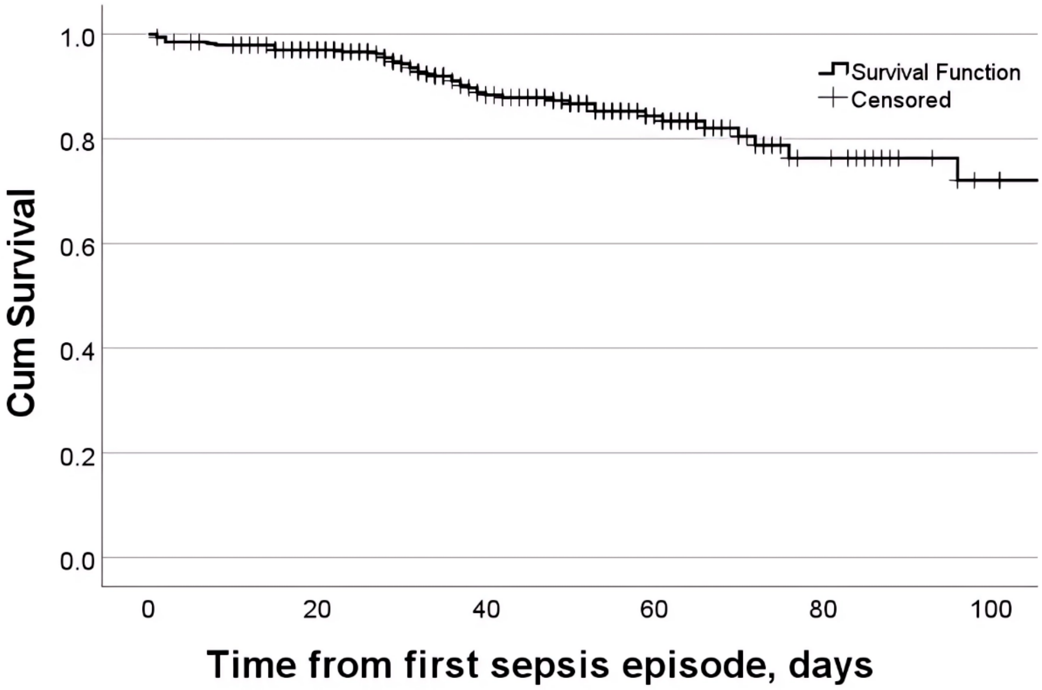

Number at risk

336 289 187 86 28 15
